# Plant-Based Diets: A Path to Ending CVD as We Know It?

**DOI:** 10.3390/nu15163608

**Published:** 2023-08-17

**Authors:** Rami Salim Najjar, Andrew T. Gewirtz

**Affiliations:** Center for Inflammation, Immunity and Infection, Institute for Biomedical Sciences, Georgia State University, Atlanta, GA 30303, USA; agewirtz@gsu.edu

Cardiovascular disease (CVD) is the leading cause of death in the United States, with roughly 700,000 CVD deaths every year [1]. Lifestyle factors account for >80% of CVD risk [2], with diet being a major disease determinant [3]. An extensive body of epidemiological studies led the American Heart Association (AHA) to conclude that diets comprised of minimally processed plant foods associate with optimal cardiovascular health, while in contrast, diets rich in animal products associate with poor cardiovascular health [4]. Such observations may explain the relatively high incidence of CVD in westernized countries, including the United States, in which animal product intake well exceeds recommended dietary recommendations (140% of recommended intakes) [5], while consumption of unrefined plant foods is severely lacking (Table 1). Our recently published literature review outlines numerous molecular mechanisms by which animal food-rich diets may drive CVD pathogenesis [6]. Conversely, while some of the benefits of plant-based diets likely simply reflect reduced consumption of animal products, a rapidly developing body of research indicates that, in fact, many plant foods have bioactive components that confer numerous benefits on cardiovascular health irrespective of the extent to which they reduce animal products. Some of these benefits are outlined in this issue.

The consumption of fiber from unprocessed foods can be considered a proxy for plant food consumption, as fiber is derived from plants. A recent rigorous systematic analysis comprising ~135 million person-years of data from Reynolds et al. [8] found that fiber intake (as well as whole grain intake) was inversely associated with CVD incidence and mortality in a dose-dependent manner. However, considering usual dietary intake data as illustrated in Table 1, it is not surprising that National Health and Nutrition Examination Survey (NHANES) data reveals that overall, in males and females, 6% of Americans meet the bare minimum recommendations for fiber intake [9]. It is important to note that consuming fiber is not merely a proxy for reducing animal product consumption. Based on compelling in vivo data we have generated, fiber is a key dietary component that can protect against metabolic disorders by positively modulating the gut microbiota [10,11]. In fact, we have illustrated previously that maternal low-fiber intake can detrimentally impact gut microbial health of offspring, increasing the propensity for obesity with an obesogenic diet, even after consumption of a higher diet in offspring [12]. Considering the major role of the gut microbiome in CVD, both in CVD protection (via short-chain fatty acid production) and, inversely, in CVD promotion (via trimethylamine N-oxide production) [13], positively regulating the gut microbiota by increasing plant food consumption is of major clinical relevance.

In addition to the beneficial effects of fiber, plants contain a wide array of polyphenols, secondary metabolites produced by plants that have profound bioactive properties. We have previously highlighted numerous molecular targets of the cardiovascular system that polyphenols could mediate [14,15,16]. These include redox and inflammatory pathways, mitochondrial function, apoptotic pathways, Ca^2+^ handling, remodeling pathways, vasodilatory pathways, the renin-angiotensin system, as well as adrenergic signaling. However, the studies assessed were performed either in vitro or in vivo in animal models utilizing single polyphenols rather than the multitude found in any one food, not even considering those found in a combination of foods. Each plant has a unique and distinct profile of polyphenols [17]. Thus, the consumption of a heterogeneous plant-based diet, as is typically the case with human eating patterns, facilitates potential synergistic effects. For example, we have found that in high-fat diet-fed mice, the consumption of blackberries and raspberries in combination was far more efficacious in improving cardiac inflammatory signaling and redox pathways compared to any one of the berries alone [18]. Indeed, Mendonça et al. [19] have demonstrated that in young-middle aged individuals, the highest quintile of polyphenol consumption was associated with a 47% lower incidence of cardiovascular events compared to the lowest quintile, while a reanalysis of the PREDIMED trial has revealed that those with the highest polyphenol intake had a 37% reduced risk of all-cause mortality [20]. Thus, polyphenols could be one of the most important CVD-protective factors of a plant-based diet; however, much more clinical work is needed on this front.

As of the time of writing, a strict plant-based diet was the only dietary intervention identified in the literature to clinically reverse atherosclerosis [21,22], treat heart failure [23,24,25,26], and improve myocardial perfusion [27]. A weight-maintaining fully plant-based diet rich in raw fruits and vegetables was found to reduce low-density lipoproteins to a degree comparable to statin treatment [28], and our prior work has demonstrated that a strict plant-based diet was able to reduce blood pressure more efficaciously than hypertensive managing drugs [29], reducing the medication burden of patients. While the results of these aforementioned works are impressive, the rigor of these investigations is mixed. While much clinical work has been done on Mediterranean diets, this dietary approach is more of a mixed diet, and while plant food intake is indeed increased, it is not necessarily a plant-based diet definitionally.

Thus, we identified a major gap in the literature, which was the impetus for the present Special Issue, “*Plant-Based Diets in CVD Prevention: Molecular Mechanisms and Biochemical Insights*”. The goal of this Special Issue is two-part: (1) expand our knowledge on the potential efficacy of plant-based diets in treating CVDs by expanding the limited scientific record; and (2) understand the molecular mechanisms by which plant-based diets could target CVD, explaining their potential efficacy. While clinical data will be the most impactful and relevant to the goals of this Special Issue, the utilization of in vitro or animal models is also recognized as a useful avenue in identifying molecular targets by which plant-based diets mediate and are therefore also welcome.

## Figures and Tables

**Table 1:** The percentage of individuals in the USA who do not meet minimum recommended dietary intakes of various plant food groups. Adapted from Krebs-Smith et al. [7]. Data utilizes both males and females ≥ 2 years of age (*n* = 16,338).

Total Fruits	Whole Fruits	Total Vegetables	Dark Green Vegetables	Orange Vegetables	Legumes	Starchy Vegetables	Other Vegetables	Whole Grains
79.6%	75.0%	88.7%	96.1%	97.5%	96.1%	60.7%	58.4%	99.3%
